# The association between Chinese visceral adiposity index and hypertension among middle-aged and older population: a cross-sectional study based on CHARLS

**DOI:** 10.3389/fcvm.2025.1664848

**Published:** 2025-10-31

**Authors:** Jialei Ma, Jun Ren, Yujia Chen, Lili Xue, Yufang Huang, Jin Qian, Yan Chen, Mudan Lu, Yaqin Zhong

**Affiliations:** ^1^ School of Public Health, Nantong University, Nantong, Jiangsu, China; ^2^ Changshu Hospital Affiliated to Soochow University, Changshu NO. 1 people’s Hospital, Changshu, China; ^3^ Public Health Department, Changshu Hospital Affiliated Hospital of Nantong University, Changshu NO. 2 people’s Hospital, Changshu, China; ^4^ Department of Cardiology, The Affiliated Huishan People’s Hospital of Xinglin College, Nantong University, Wuxi Huishan District People’s Hospital, Wuxi, China; ^5^ Wuxi Maternity and Child Health Care Hospital, Affiliated Women’s Hospital of Jiangnan University, Wuxi, China

**Keywords:** Visceral Adiposity Index, hypertension, middle-aged and older adults, cross-sectional study, risk assessment

## Abstract

**Background:**

In recent years, obesity has become a serious public health issue. This study aims to investigate the association between the Chinese Visceral Adiposity Index (CVAI) and hypertension among the middle-aged and older population in China.

**Methods:**

Data from the China Health and Retirement Longitudinal Study (CHARLS) 2015 wave were used. A 3-knot restricted cubic spline (RCS) was employed to analyze the dose-response relationship between CVAl and hypertension. Logistic regression model was used to explore the association between CVAI and hypertension, adjusting for confounding factors including age, sex, education level, smoking, alcohol consumption, body mass index (BMI), diabetes, hyperlipidemia, and heart disease.

**Results:**

A total of 8,787 individuals were included in the study with a hypertension prevalence of 27.89%. A significant association between CVAI and hypertension was observed. Compared to those in the low CVAI category, hypertension was significantly associated with individuals in the high CVAI category in logistic regression (OR adjusted for confounding factors =1.967, 95% CI: 1.781, 2.172). The results showed that the risk of hypertension significantly increased with higher CVAI (trend test *p* < 0.001). Additionally, subgroup analyses demonstrated a stronger association between CVAI and hypertension among women (OR = 2.5, 95% CI: 2.18–2.88; *P* for interaction <0.001) and non-smokers (OR = 2.44, 95% CI: 2.14–2.78; *P* for interaction = 0.001).

**Conclusion:**

CVAI may therefore potentially serve as a useful biomarker for identifying individuals at higher risk, and controlling visceral adiposity accumulation may be a potential target for the prevention and treatment of hypertension.

## Introduction

Hypertension, a prevalent chronic cardiovascular disease, is a significant public health challenge worldwide (1, 2). According to World Health Organization（WHO） statistics, there are over 100 billion patients with hypertension globally, and the number is expected to rise (3–5). Hypertension is not only a cardiovascular disease but also a major risk factor for many other conditions, including coronary heart disease, stroke, and chronic kidney disease. It significantly increases the global economic burden and threatens human lives. In China, the prevalence of hypertension is alarming high. Recent studies indicate that more than 25% of adults in China have hypertension, and the prevalence significantly increases with age (6). Therefore, investigating the risk factors of hypertension and underlying molecular mechanisms is crucial for the prevention and treatment of hypertension.

Recently, with the development of the economy and society, obesity has become another serious public health problem. There is a growing awareness of the different patterns of obesity (7, 8). Central obesity, characterized by visceral adiposity accumulation, has been demonstrated to be more harmful to human health. Visceral adiposity refers to fat wrapping around abdominal organs (9, 10). Compared to subcutaneous fat, visceral adiposity has higher metabolic activity, releasing various bioactive substances, including inflammatory factors, free fatty acids, and others. These molecules can directly regulate hypertension and induce insulin resistance, chronic inflammation, and endothelial dysfunction, eventually leading to hypertension. The Chinese Visceral Adiposity Index (CVAI), a quantitative measure of visceral adiposity content, has been widely used in research on obesity-related diseases (11, 12). However, research on the correlation between CVAI and hypertension in the Chinese middle-aged and older population remains relatively limited.

The China Health and Retirement Longitudinal Study (CHARLS) is a nationally representative large-scale longitudinal survey designed to collect multi-dimensional data on health, economic status, and social behavior among the Chinese population aged 45 and above (13).In this study, we conducted a cross—sectional analysis based on the CHARLS data of 2015 wave, aiming to explore the relationship between the Chinese Visceral Adiposity Index (CVAI) and hypertension, so as to provide a basis for further in—depth research on the association between obesity and hypertension.

## Methods

### Study population

**Figure 1:**
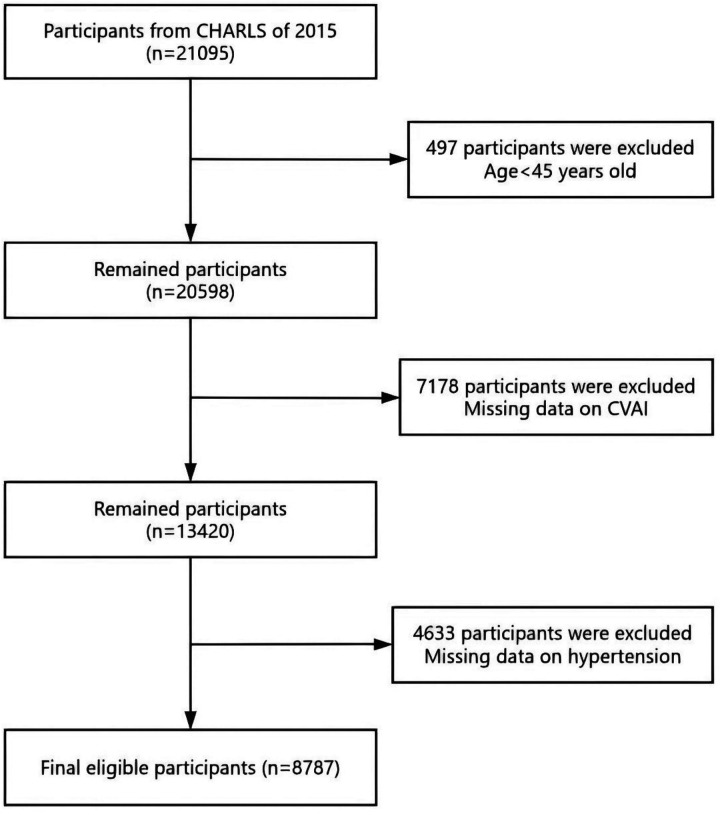
The flowchart indicates the participants of the study.

The CHARLS project aimed to provide a high-quality microdata set representing households and individuals, facilitating research on population aging and interdisciplinary studies related to aging. The survey covered a wide range of topics, including demographic information, family structure, intergenerational transfers, health status, medical care and insurance, employment, income, expenditures, and assets. The national baseline survey of CHARLS was launched in 2011, employing a multi-stage probability proportional to size (PPS) sampling method. CHARLS was an ongoing longitudinal survey, with follow-up waves conducted every 2–3 years. The survey sample included 450 villages, 150 counties, and 28 provinces, covering over 17,000 individuals from approximately 10,000 households. Data collection involved face-to-face interviews conducted in participants' homes using computer-assisted personal interviewing (CAPI) technology. The datasets were publicly available and could be accessed from the CHARLS homepage at http://charls.pku.edu. cn/en. This study utilized data from the wave 3 in 2015 of CHARLS, which includes detailed health examination indicators and lifestyle information. We initially included 21,095 individuals. The exclusion criteria were as follows: (1) individuals aged below 45 (*n* = 497); (2) individuals with incomplete data on CVAI (*n* = 7,178); (3) individuals with incomplete data on hypertension (*n* = 4,633); and eventually, 8,787 participants were included, and the screening process is illustrated in Figure 1.

### CVAI measurement

CVAI was a comprehensive indicator that integrates anthropometric measurements and functional parameters to assess visceral adiposity distribution. Following the studies of Amato (14) and Han (15), a specific formula incorporating waist circumference (WC), body mass index (BMI), triglycerides (TG), and high-density lipoprotein cholesterol (HDL-C) was used to calculated CVAI. Due to differences in adiposity distribution and metabolism between men and women, the CVAI formula was tailored for each gender to improve accuracy. CVAI was calculated using the following formulas:

CVAI (male) = −267.93 + 0.68 × age (years) + 0.03 × BMI (kg/m^2^) + 4.00 × WC (cm) + 22.00 × LgTG (mmol/L) - 16.32 × HDL-C (mmol/L)

CVAI (female) = −187.32 + 1.71 × age (years) + 4.23 × BMI (kg/m^2^) + 1.12 × WC (cm) + 39.76 × LgTG (mmol/L) - 11.66 × HDL-C (mmol/L)

BMI is expressed in kg/m^2^, calculated as weight (kg) divided by height squared (m^2^).

### Definition of hypertension

Hypertension was determined using both self-reported medical history and blood pressure measurements. Hypertension was defined as either: (1) a self-reported physician's diagnosis of hypertension; or (2) measured systolic blood pressure ≥140 mmHg and/or diastolic blood pressure ≥90 mmHg, based on the average of three readings; or (3) current use of antihypertensive medication.

### Confounding factors

The CHARLS working group used computer-assisted personal interviewing (CAPI) technology, and all physical and biochemical measurements were conducted by trained professionals using standardized protocols. Demographic and lifestyle information were collected through structured household interviews conducted by trained surveyors, including age, sex (female, male), residence (rural, urban), education level (illiteracy, primary school, middle school, high school and above), smoking status, drinking status. Health conditions and medical history were obtained through self-reported questionnaires and medical history interviews. Participants were asked if they had ever been diagnosed with dyslipidemia, diabetes mellitus (DM), heart diseases by a doctor. Participants also reported whether they were currently taking lipid-lowering medications. Blood samples were collected by trained healthcare professionals at participants' homes, and the samples were analyzed in certified laboratories. Trained medical professionals conducted standardized physical examinations to collect anthropometric and blood pressure measurements. Detailed methodologies are accessible on the CHARLS website (http://charls.pku.edu.cn/).

### Statistics

The baseline characteristics of the study population were calculated, including the mean and standard deviation for continuous variables and the frequency and percentage for categorical variables. The differences in CVAI and other covariates between the hypertension and non-hypertension groups were compared using *t*-tests or chi-square tests. A 3-knot restricted cubic spline (RCS) plot was utilized to explore the nonlinear relationship between CVAI and hypertension. Specifically, we used three knots placed at the 10th, 50th, and 90th percentiles of the CVAI distribution, which is a commonly recommended approach in epidemiological studies to balance model flexibility and stability. The aim was to establish the cutoff point using the RCS curve, followed by conducting a logistic regression analysis anchored on this threshold. A logistic regression model was conducted to analyze the association between CVAI and hypertension, adjusting for potential confounding factors. The results were expressed as odds ratios (ORs) with 95% confidence intervals (CIs). Subgroup analyses were conducted to explore the heterogeneity in the association between CVAI and hypertension. While our subgroup analyses suggested potential effect modifications by factors such as age, sex, and smoking status, it is important to acknowledge the issue of multiple testing. Conducting numerous statistical comparisons increases the probability of Type I error, where an association is falsely deemed significant. The CHARLS study was approved by the Ethics Committee of Peking University, and all participants provided informed consent. This study used publicly available anonymized data, which did not require additional ethical review. All statistical analyses were conducted using spss27.0, Stata 17.0, and R statistical software, with a significance level set at *P* < 0.05.

## Results

### General characteristics of participants

The study included a total of 8,787 individuals, with 2,626 (27.89%) having hypertension and 6,161 (70.11%) without hypertension (Table 1). The results indicated that the incidence of hypertension among the middle-aged and older adults varied based on several factors, including age, gender, education, smoking, drinking, dyslipidemia, diabetes mellitus (DM), use of lipid-lowering drugs, heart diseases, fasting blood glucose levels, creatinine, uric acid, triglycerides (TG), total cholesterol (TC), low-density lipoprotein (LDL), weight, body mass index (BMI), waist circumference, systolic blood pressure (SBP), diastolic blood pressure (DBP), and the Chinese visceral adiposity index (CVAI). The differences among these factors were statistically significant (*p* < 0.05). The average age of the entire sample was 61.46 ± 9.19 years, with hypertensive individuals being significantly older (63.98 ± 9.26 years) compared to non-hypertensive individuals (60.39 ± 8.95 years, *p* < 0.001). There were 1,334 females with hypertension (27.91%) and 3,445 without hypertension (72.09%). Among the middle-aged and older adults living in rural areas, 4,763 (70.06%) were in the non-hypertensive group, while 2,035 (29.94%) were in the hypertensive group. Among middle-aged and older adults with a high school education or higher, 263 (27.86%) were diagnosed with hypertension, while 681 (72.14%) were non-hypertension. Of those with hypertension, 1,139 (31.84%) were smokers, and 1,487 (28.54%) were non-smokers. In terms of alcohol consumption, 2,068 (68.57%) of the middle-aged and older adults in the non-hypertensive group consumed alcohol, compared to 948 (31.43%) in the hypertensive group. Regarding comorbidities, among middle-aged and older adults with hypertension, 406 individuals (38.85%) had dyslipidemia, 235 (40.80%) had diabetes mellitus (DM), and 421 (36.64%) had heart diseases. Among participants using lipid-lowering drugs, 600 (61.22%) were in the non-hypertensive group, whereas 380 (38.78%) were in the hypertensive group. Biochemically, hypertensive individuals had significantly higher levels of fasting blood glucose (104.25 ± 33.41 mg/dl), creatinine (0.82 ± 0.25 mg/dl), uric acid (5.10 ± 1.47 mg/dl), total cholesterol (188.57 ± 37.26 mg/dl), triglycerides (148.82 ± 95.03 mg/dl), and low-density lipoprotein (LDL) (105.10 ± 29.56 mg/dl), compared to those without hypertension. Other physical measurements showed that hypertensive individuals had a significantly higher weight (61.90 ± 12.18 kg), BMI (24.79 ± 4.04 kg/m^2^), and waist circumference (88.72 ± 11.52 cm). Systolic blood pressure (SBP) and diastolic blood pressure (DBP) were also significantly higher in hypertensive individuals (152.38 ± 15.58 mmHg and 86.45 ± 11.18 mmHg, respectively). The Chinese visceral adiposity index (CVAI) was significantly higher in hypertensive individuals (122.30 ± 40.06) compared to non-hypertensive individuals (104.54 ± 38.72, *p* < 0.001), suggesting a strong association between CVAI and hypertension.

**Table 1:** Baseline characteristics of the study population stratified by hypertension status.

Variable	Total (*n* = 8,787)	Hypertension (*n* = 2,626)	Non-hypertension (*n* = 6,161)	*P*-value
Age	61.46 ± 9.19	63.98 ± 9.26	60.39 ± 8.95	<0.001
Sex,%				<0.001
Female	4,779 (54.39)	1,334 (27.91)	3,445 (72.09)	
Male	4,008 (45.61)	1,292 (32.24)	2,716 (67.76)	
Residence, %				0.849
Rural	6,798 (77.36)	2,035 (29.94)	4,763 (70.06)	
Urban	1,989 (22.64)	591 (29.71)	1,398 (70.29)	
Education, %				<0.001
Illiteracy	2,248 (25.58)	774 (34.43)	1,474 (65.57)	
Primary school	3,695 (42.05)	1,076 (29.12)	2,619 (70.88)	
Middle school	1,900 (21.63)	513 (27.00)	1,387 (73.00)	
High school and above	944 (10.74)	263 (27.86)	681 (72.14)	
Smoking, %				0.001
No	5,210 (59.29)	1,487 (28.54)	3,723 (71.46)	
Yes	3,577 (40.71)	1,139 (31.84)	2,438 (68.16)	
Drinking, %				0.022
No	5,771 (65.68)	1,678 (29.08)	4,093 (70.92)	
Yes	3,016 (34.32)	948 (31.43)	2,068 (68.57)	
Dyslipidemia, %				<0.001
No	7,742 (88.11)	2,220 (28.67)	5,522 (71.33)	
Yes	1,045 (11.89)	406 (38.85)	639 (61.15)	
DM, %				<0.001
No	8,211 (93.44)	2,391 (29.12)	5,820 (70.88)	
Yes	576 (6.56)	235 (40.80)	341 (59.20)	
Lipids lowering druga, %				<0.001
No	7,807 (88.85)	2,246 (28.77)	5,561 (71.23)	
Yes	980 (11.15)	380 (38.78)	600 (61.22)	
Heart diseases, %				<0.001
No	7,638 (86.92)	2,205 (28.87)	5,433 (71.13)	
Yes	1,149 (13.08)	421 (36.64)	728 (63.36)	
Fasting blood glucose (mg/dl)	100.89 ± 30.89	104.25 ± 33.41	99.45 ± 29.63	<0.001
Creatinine (mg/dl)	0.80 ± 0.31	0.82 ± 0.25	0.80 ± 0.33	0.006
Uric acid (mg/dl)	4.92 ± 1.40	5.10 ± 1.47	4.85 ± 1.36	<0.001
TG (mg/dl)	138.27 ± 87.33	148.82 ± 95.03	133.77 ± 83.43	<0.001
TC(mg/dl)	184.63 ± 36.46	188.57 ± 37.26	182.95 ± 35.99	<0.001
HDL (mg/dl)	51.43 ± 11.66	51.56 ± 12.30	51.37 ± 11.38	0.490
LDL (mg/dl)	102.98 ± 28.96	105.10 ± 29.56	102.07 ± 28.66	<0.001
Height (m)	157.94 ± 8.53	157.76 ± 8.89	158.03 ± 8.36	0.194
Weight (kg)	59.92 ± 11.56	61.90 ± 12.18	59.08 ± 11.18	<0.001
BMI (Kg/m^2)^	23.96 ± 3.88	24.79 ± 4.04	23.60 ± 3.76	<0.001
Waist (cm)	86.09 ± 11.51	88.72 ± 11.52	84.96 ± 11.32	<0.001
SBP (mmHg)	129.08 ± 20.01	152.38 ± 15.58	119.15 ± 11.73	<0.001
DBP (mmHg)	75.89 ± 11.53	86.45 ± 11.18	71.39 ± 8.28	<0.001
CVAl	109.85 ± 39.96	122.30 ± 40.06	104.54 ± 38.72	<0.001

Data are presented as mean ± standard deviation (SD) for continuous variables and n(%)for categorical variables.

DM, Diabetes mellitus; TC, Total cholesterol; TG, Triglycerides; HDL, High-density lipoprotein cholesterol; LDL, Low-density lipoprotein cholesterol. *P*-values are derived from *t*-tests for continuous variables and chi-square tests for categorical variables.

### Dose-response relationship between the CVAl and hypertension

**Figure 2:**
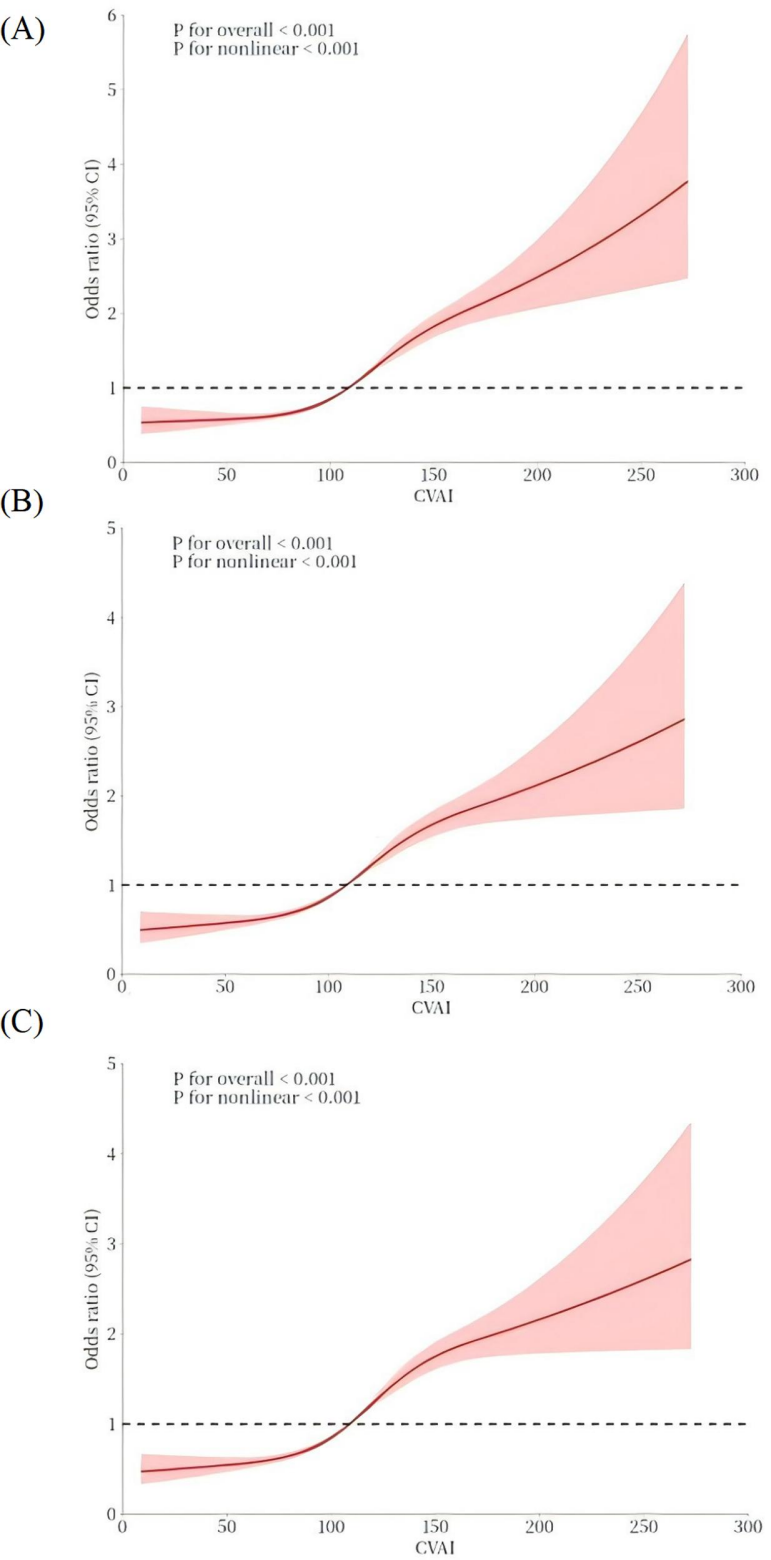
The dose–response relationship between CVAI and hypertension. A 3-knot RCS was employed to fit the dose–response relationship between CVAI and hypertension. **(A)** Model 1: no covariates were adjusted. **(B)** Model 2: age, sex. **(C)** Model 3: age, sex, education, residence, smoking, alcohol consumption, diabetes, hyperlipidemia, heart disease, and lipid-lowering medication use. The red solid line represents the curve fitting between variables, and the shaded area indicates the 95% CI of the fit.

In addition, a 3-knot RCS was employed to model the dose-response relationship between CVAl and elevated hypertension, as depicted in Figure 2. The CVAI was plotted as the horizontal coordinate and the odds ratio (OR) was plotted as the vertical coordinate. The results, after adjusting for potential covariates, indicated that higher levels of CVAI cor-responded with an increasing risk of hypertension (nonlinear *P* < 0.001). The corresponding inflection point value was 108.88.

The relationship between CVAI and hypertension is displayed in Table 2. After controlled for potential covariates in Model 3, the findings indicated a significant positive relationship with an OR of 1.010 (95% CI: 1.008, 1.011). The RCS was applied to examine the nonlinear relationship between CVAI and hypertension. An inflection point value of 108.88 was derived (see Figure 2C), which was then used as a cutoff point to classify the CVAI as a dichotomous variable for logistic regression. In Model 1, the OR for the upper category in comparison with the lower category was 2.189 (95% CI: 1.993, 2.405). In Model 2, after controlled for age and gender, the OR for the higher category increased to 2.019 (95% CI: 1.834, 2.223). In model 3, multiple confounding factors were controlled, including age, sex, education, residence, smoking, alcohol consumption, diabetes, hyperlipidemia, heart disease, and lipid- lowering medication use. The OR was 1.967 (95% CI: 1.781, 2.172), indicating that the relationship between higher CVAI and hypertension remained statistically significant. The trend analysis confirmed a consistent positive correlation between the two categories (P for trend < 0.001).

**Table 2:** Relationship between CVAI and hypertension.

Variable	Model 1		Model 2		Model 3	
	OR (95%CI)	*P*-value	OR (95%CI)	*P*-value	OR (95%CI)	*P*-value
CVAI						
Continuous	1.011 (1.010, 1.013)	<0.001	1.010 (1.009, 1.011)	<0.001	1.010 (1.008, 1.011)	<0.001
CVAI < 108.88	1.0		1.0		1.0	
CVAI ≥ 108.88	2.189 (1.993, 2.405)	<0.001	2.019 (1.834, 2.223)	<0.001	1.967 (1.781, 2.172)	<0.001
*P* for trend	<0.001		<0.001		<0.001	

Model 1: no covariates were adjusted. Model 2: age, sex. Model 3: age, sex, education, residence, smoking, alcohol consumption, diabetes, hyperlipidemia, heart disease, and lipid-lowering medication use.

CVAI, Chinese visceral adiposity index; OR odds ratio; CI, confidence interval.

### Subgroup analyses

**Figure 3:**
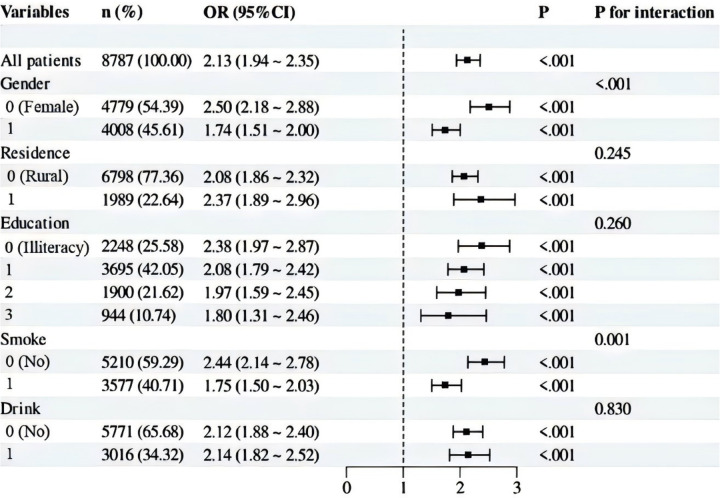
Subgroup analysis of CVAI and hypertension.

Our subgroup analysis utilized stratification variables such as sex (Male vs. Female), residence (Rural vs. Urban), education (Illiteracy vs. Primary school vs. Middle school vs. High school and above), smoke (Yes vs. No), and drinking status (Yes vs. No). The results were visually presented using a forest plot. As presented in Figure 3, these analyses revealed that these factors influenced the relationship between the CVAI and hypertension. Specifically, a positive association between CVAI and hypertension was observed across all subgroups. This association appeared to be stronger within specific subgroups: such as women (*P* for interaction < 0.001), and non-smokers (*P* for interaction = 0.001). In contrast, residence, education, drinking status did not appear to have a substantial impact on the relationship (*P* for interaction >0.05). Overall, the test for interaction between subgroups indicated that the relationship between CVAI and hypertension varied significantly across different subgroups.

## Discussion

In this study, we conducted a cross-sectional analysis among middle-aged and older adults in China (aged over 45 years) to systematically investigate the association between CVAI and hypertension. We found a positive correlation between Chinese visceral adiposity accumulation and the occurrence of hypertension, suggesting that controlling visceral adiposity accumulation may be a potential target for the prevention and treatment of hypertension. Besides, the dose-response analysis revealed a nonlinear association between CVAI and hypertension risk, with a notable inflection point at 108.88, indicating a critical threshold where the risk of hypertension markedly increases, which might be helpful in clinical practice and for heathy police makers. However, due to the inherent limitations of cross-sectional studies, our study cannot establish a causal relationship between CVAI and hypertension, therefore, further prospective studies are needed to confirm these findings.

From biological mechanisms, we infer that visceral adiposity could contribute to vascular dysfunction from the aspects, including promoting inflammation, oxidative stress, and adipokine imbalance. Blood vessels are primarily composed of endothelium and smooth muscle. As the most abundant cellular component in blood vessels, smooth muscle dysfunction can significantly impair vascular function, contributing to the development and progression of various chronic cardiovascular diseases, including hypertension (16, 17). From a molecular mechanism perspective, numerous studies have confirmed that visceral adiposity accumulation can lead to vascular smooth muscle dysfunction. First, unlike subcutaneous fat, visceral adiposity has higher secretory activity and can release various inflammatory mediators, including TNF-α, IL-6, and IL-8 (18, 19). These circulating inflammatory mediators may induce inflammation in smooth muscle, eventually leading to vascular fibrosis and calcification, which results in impaired vasodilation. Furthermore, oxidative stress plays a crucial role in vascular sclerosis. In addition to increasing systemic inflammation levels, visceral adiposity also contributes to the accumulation of reactive oxygen species (ROS) (20–23). Vascular endothelial cells also play a crucial role in maintaining vascular homeostasis. Studies have shown that reducing visceral adipose tissue (VAT) can significantly improve NO production, reduce inflammation levels, and enhance vascular dilation function. Recent studies have shown that inflammatory responses play a crucial role in the development and progression of cardiovascular diseases, particularly in VAT and perivascular adipose tissue (PVAT) (21). For example, a study on hereditary hypertriglyceridemic (HHTg) rats found that sex differences significantly influence inflammatory responses and hematological status, thereby affecting cardiovascular disease risk. In this study, female HHTg rats exhibited more pronounced hypertriglyceridemia and a procoagulant state, whereas male rats showed higher non-fasting blood glucose levels and serum leptin levels. Additionally, ICAM-1 gene expression was increased in the aorta of male HHTg rats, while TNF*α* gene expression was lower in the aorta of female rats, suggesting that females had a relatively milder inflammatory response (24–26). Due to the multifaceted and multi-mechanistic damage caused by visceral adiposity to blood vessels, its detrimental effects are particularly strong. In this study, we conducted a detailed subgroup analysis, revealing a significant association between visceral adiposity and hypertension across different populations. Notably, this association was stronger in women and non-smokers, with statistically significant differences. One possible explanation is that women, particularly postmenopausal women, may experience hormonal changes that influence fat distribution and cardiovascular risk, thereby amplifying the effect of visceral fat on hypertension. Similarly, in non-smokers, the absence of smoking-related vascular damage may make the hypertensive effect of visceral adiposity more discernible.

Actually, previous clinical studies already explored the association between visceral adiposity and hypertension. A long-term MRI-based follow-up study demonstrated that baseline VAT area (VAT cm^2^) and the proportion of VAT to total abdominal adiposity (VAT%) were significantly associated with metabolic syndrome, hypertension, and diabetes status (27). The study further indicated that while VAT cm^2^ and VAT% performed similarly in predicting metabolic syndrome and hypertension, VAT% was more advantageous in assessing lipid status, whereas VAT cm^2^ better reflected inflammation and blood glucose status. This aligns with our findings, suggesting that CVAI, as a comprehensive index, may incorporate the clinical significance of both VAT cm^2^ and VAT%, thereby offering unique value in predicting hypertension and other cardiometabolic risks. Additionally, the MRI study found that after 18 months of lifestyle intervention, reductions in VAT cm^2^ and VAT% were significantly correlated with decreases in triglycerides, HbA1c, ferritin, and liver enzymes, as well as increases in HDL-c levels. These results further support the beneficial role of visceral adiposity reduction in improving metabolic health and suggest that CVAI may serve as a potential indicator for evaluating the effectiveness of lifestyle interventions (28, 29). However, MRI is not a routine examination, as it requires a relative high cost and technical expertise. Our study on the relationship between CVAI and hypertension provides a more accessible approach to understanding this association of abdominal adiposity and hypertension. Notably, we identified a critical inflection point at 108.88, beyond which the correlation between CVAI and hypertension becomes more pronounced. This finding may contribute to future hypertension prevention and management strategies.

This study has several limitations. First, the wave 3 of CHARLS was used in the present study, it can only reveal the association between CVAI and hypertension but cannot establish a causal relationship. CHARLS collects data from the same group of samples at multiple time points, the cross-sectional nature of our analysis is due to the unavailability of comparable data from other years. The reason to utilize the 2015 dataset is because blood pressure measurements were not continued for participants in subsequent waves. Second, this study did not examine the dynamic changes in CVAI and their impact on hypertension risk. Future longitudinal studies should investigate the trends in CVAI over time and their relationship with hypertension development to further validate its predictive value. Third, this study mainly relied on the indirect calculation of CVAI and did not directly measure VAT volume or vascular function indicators (such as flow-mediated dilation or endothelial function biomarkers). Additionally, although we adjusted for multiple confounding factors in the multivariable regression analysis, unmeasured or residual confounders may still exist, potentially affecting the relationship between CVAI and hypertension. Finally, since CHARLS primarily focuses on middle-aged and older adults in China, the findings may not be generalizable to other age groups or ethnic populations. Future studies should extend to diverse populations to assess the generalizability of CVAI.

## Conclusion

This study, based on the CHARLS database, systematically explored the relationship between CVAI and hypertension in the middle-aged and older population in China. The results show a significant positive association between CVAI and hypertension, which is consistent across subgroup analysis of gender, and smoking status. Although this is a cross-sectional study and causality cannot be determined, the findings suggest that visceral fat accumulation may be an important risk factor for hypertension. Controlling visceral adiposity accumulation may provide a new strategy for the prevention and treatment of hypertension.

## Data Availability

The original contributions presented in the study are included in the article/Supplementary Material, further inquiries can be directed to the corresponding authors.
